# Novel lncRNA Signature (UFC1/PTENP1) as a Molecular Biomarker for the Diagnosis and Prognosis of Hepatocellular Carcinoma in an Egyptian Cohort

**DOI:** 10.3390/cimb48040360

**Published:** 2026-03-29

**Authors:** Marwa Hassan, Lobna Abdelsalam, Amal Kotb Behery, Rania Fathy Elnahas

**Affiliations:** 1 Immunology Department, Theodor Bilharz Research Institute, Giza 12411, Egypt; 2 Human Genetics Department, Medical Research Institute, Alexandria University, Alexandria 21561, Egypt

**Keywords:** biomarker, diagnosis, Egyptian cohort, fibrosis, hepatocellular carcinoma, long non-coding RNA (lncRNA), prognosis, PTENP1, UFC1

## Abstract

Long non-coding RNAs (lncRNAs) are key regulators of gene expression and play critical roles in cancer-related signaling networks. Dysregulation of antagonistic lncRNAs may contribute to hepatocarcinogenesis and disease progression. This study investigated the clinical significance and predictive value of two biologically antagonistic lncRNAs, UFC1 and PTENP1, as circulating biomarkers for hepatocellular carcinoma (HCC) in an Egyptian cohort. Expression levels of these lncRNAs were quantified in 100 HCC patients and 100 age- and sex-matched healthy controls. UFC1 was significantly upregulated (~2.9-fold), while PTENP1 was markedly downregulated (~4-fold) in HCC patients, with a strong inverse correlation (r = −0.609, *p* < 0.001). Both lncRNAs demonstrated higher diagnostic accuracy compared to alpha-fetoprotein (AFP); combining them with AFP further enhanced overall performance. UFC1 expression was increased progressively with advancing fibrosis grade and Barcelona Clinic Liver Cancer (BCLC) stage, while PTENP1 levels diminished with BCLC stage. Logistic regression confirmed UFC1 as an independent risk factor and PTENP1 as a protective factor for HCC. In conclusion, the blood-based UFC1/PTENP1 panel exhibits promising diagnostic accuracy and is associated with disease severity, surpassing AFP. Their fibrosis-associated dysregulation suggests a role in early hepatocarcinogenesis. This antagonistic lncRNA signature represents a potential, non-invasive tool for HCC detection and risk stratification, meriting further clinical validation.

## 1. Introduction

Hepatocellular carcinoma (HCC) is the predominant primary liver malignancy and is the third greatest cause of cancer-related deaths worldwide. Its incidence continues to rise, largely driven by persistent infections with hepatitis B (HBV) and C (HCV) viruses, alcohol intake, and the growing prevalence of metabolic dysfunction–associated fatty liver disease [1]. Notwithstanding advances in therapeutic strategies, the prognosis of HCC remains poor, primarily due to late diagnosis at advanced, often untreatable stages, and the high rate of tumor recurrence [2]. Therefore, early identification and precise risk stratification remain critical challenges in cancer management [3,4,5].

Current surveillance paradigms rely mainly on a combination of imaging modalities and measurement of serum alpha-fetoprotein (AFP). However, this approach possesses considerable constraints. Abdominal ultrasound may lack sufficient sensitivity for detecting small, early-stage tumors. CT raises concerns regarding exposure to radiation and possible adverse reactions to iodinated contrast media. MRI, although extremely sensitive, is limited by its cost and restricted availability [6]. More importantly, AFP exhibits suboptimal specificity and sensitivity, with elevated levels also present in benign chronic liver conditions, and a substantial proportion of HCC patients have normal AFP levels [7]. This diagnostic gap underscores an urgent need for novel, highly sensitive, and specific non-invasive biomarkers to enable earlier detection, refine prognostic assessment, and optimize patient stratification.

In this context, the exploration of the non-coding genome has opened new avenues for promising molecular players and biomarkers in oncology [8,9]. Defined as transcripts longer than 200 nucleotides that lack protein-coding potential, long non-coding RNAs (lncRNAs) are key regulators of gene expression at the epigenetic, transcriptional, and post-transcriptional levels. Consequently, they are increasingly implicated in critical cellular processes, including proliferation, apoptosis, differentiation, and immune evasion [10]. Their dysregulation is a hallmark of various cancers, and owing to their remarkable tissue- and disease-specificity, along with stability in bodily fluids, lncRNAs offer immense potential as candidates for liquid biopsy-based cancer diagnostics [11].

Among the numerous lncRNAs associated with malignancies, ubiquitin-fold modifier conjugating enzyme 1 (UFC1) and phosphatase and tensin homolog pseudogene 1 (PTENP1) represent two biologically antagonistic regulators of tumor behavior. UFC1 has been identified as an oncogenic lncRNA that promotes tumor progression by sequestering tumor-suppressive miRNAs and activating oncogenic signaling pathways, including Wnt/β-catenin [12,13]. In contrast, PTENP1 is a tumor-suppressive pseudogene that preserves PTEN mRNA by competitively binding PTEN-targeting miRNAs. Loss of PTENP1 disrupts the PTEN/PI3K/AKT signaling axis, leading to enhanced cellular proliferation, survival, and tumor progression [14]. However, the diagnostic and prognostic significance of UFC1 and PTENP1, particularly in relation to HCC and its clinical characteristics, remains incompletely understood.

Therefore, the present study was designed to evaluate the expression profiles of circulating UFC1 and PTENP1 in HCC patients compared to matched healthy controls in an Egyptian cohort, and to investigate their associations with biochemical parameters and clinicopathological features, including liver fibrosis stage and clinical disease progression. Additionally, we assessed their diagnostic accuracy and predictive performance for HCC, both individually and against the standard biomarker AFP. Elucidating the clinical relevance and prognostic value of these lncRNAs may provide insights into their utility as non-invasive tools that could improve the clinical management of HCC.

## 2. Materials and Methods

### 2.1. Study Design and Participants

This case–control study was conducted on subjects recruited from the Theodor Bilharz Research Institute (TBRI), Giza, Egypt. The study included a total of 200 participants, consisting of 100 patients with HCV-related HCC and 100 healthy controls matched for age, sex, and socioeconomic position, all of whom exhibited no signs of liver disease or other malignancies.

The diagnosis of HCC was established based on clinical evaluation, radiological findings, and laboratory investigations. Patients across all tumor stages were included and classified according to the Barcelona Clinic Liver Cancer (BCLC) staging system. Exclusion criteria involved co-infection with HBV or HIV, other causes of chronic liver disease, metastatic liver tumors, and a prior history of other primary cancers, chemotherapy, or transplantation.

The study protocol was approved by the Ethics Committee of the Medical Research Institute (IRB00010526, E/C. S/N. T78/2024), Alexandria University, and was carried out in accordance with the Declaration of Helsinki.

Clinical and laboratory data were collected, including detailed personal and clinical history, with a focus on cirrhosis, viral hepatitis infection, and other relevant comorbidities. Routine laboratory investigations were recorded, including complete blood count (CBC), complete liver function panel (alanine aminotransferase (ALT), aspartate aminotransferase (AST), alkaline phosphatase (ALP), albumin, and total bilirubin), prothrombin concentration (PC), serum creatinine, fasting glucose, AFP, hepatitis B and C serology, and quantitative HCV RNA. Radiological findings from abdominal ultrasound, with the assessment of fibrosis and hepatic steatosis, were also documented.

### 2.2. Analysis of lncRNAs Expression

Total RNA was isolated from whole blood samples stored at −80 °C. RNA extraction was performed using the High Pure RNA Isolation Kit (ROCHE, Mannheim, Germany, Cat. No. 11858882001), following the manufacturer’s protocol. RNA concentration and purity were assessed spectrophotometrically using a NanoDrop instrument (Thermo Fisher Scientific, Waltham, MA, USA), with an A260/A280 ratio between 1.8 and 2.0 considered acceptable. First-strand cDNA was synthesized from 1 µg of total RNA using the Transcriptor First Strand cDNA Synthesis Kit (ROCHE, Mannheim, Germany, Cat. No. 04896882001).

The expression levels of UFC1 and PTENP1 were quantified by qPCR using the FastStart DNA Master SYBR Green I kit (ROCHE, Mannheim, Germany, Cat. No. 04887301001) on a ROCHE LightCycler^®^ 2.0 real-time PCR instrument (ROCHE, Mannheim, Germany). All reactions were conducted in duplicate. The thermal cycling conditions comprised: initial activation at 95 °C for 10 min, followed by 40 cycles of denaturation at 95 °C for 15 s, annealing at 60 °C for 30 s, and extension at 72 °C for 30 s. A melting curve analysis was done after amplification to verify the specificity of the PCR products. The primer sequences used were: UFC1: F: 5′-TCCAACCTGAGTGACATAGCGA-3′, R: 5′-CTGACCTCCAACTCCAACGAAT-3′, PTENP1: F: 5′-TCTCTCATCTCCCTCGCCTGA-3′, R: 5′-AGCCGTGATGGAAGTTTGA-3′, and GAPDH: F: 5′-CCGGGAAACTGTGGCGTGATGG-3′, R: 5′-AGGTGGAGGAGTGGGTGTCGCT-3′. Relative gene expression was calculated using the comparative CP (ΔΔCP) method, with normalization to GAPDH expression.

### 2.3. Statistical Analysis

Data were analyzed using SPSS software (version 25; IBM Corp., Chicago, IL, USA). The normality of continuous data distribution was assessed using the Shapiro–Wilk test. Continuous variables were presented as mean ± standard error (SE) or median (interquartile range, IQR), as appropriate. Categorical variables were expressed as frequencies and percentages. Comparisons between the two groups were performed using the independent samples *t*-test or Mann–Whitney U test for continuous variables, and the Chi-square (χ^2^) test for categorical variables. The diagnostic accuracy of the lncRNAs and AFP was evaluated by constructing Receiver Operating Characteristic (ROC) curves. Spearman’s rank correlation coefficient (r) was employed to assess associations between lncRNA expression and clinical parameters. The relationship between lncRNA expression levels and disease stages (fibrosis and BCLC) was analyzed using one-way ANOVA followed by post hoc LCD test. Outlier analysis was performed using the ROUT method (Q = 1%), and analyses were conducted to confirm that the exclusion of outliers did not alter the overall statistical significance of the findings. Logistic regression analysis was performed to identify independent predictors of HCC status. A two-tailed *p*-value of <0.05 was deemed statistically significant for all analyses.

## 3. Results

### 3.1. Demographic Characteristics of the Study Population

A total of 100 patients with confirmed HCC and 100 age-, sex-, and socioeconomic-matched healthy controls were enrolled in the current study. The HCC group comprised 65 males and 35 females, aged 22 to 64 years old, with body mass index (BMI) values ranging from 17.58 to 34.16 kg/m^2^. The control group included 70 males and 30 females, aged between 23 and 59 years, with BMI values ranging from 17.20 to 33.10 kg/m^2^. Comparative statistical analysis demonstrated no significant differences between HCC patients and controls in terms of age, sex distribution, or BMI (*p* > 0.05 for all comparisons), confirming adequate demographic matching between the groups (Table 1). This comparability minimizes potential confounding effects, enabling subsequent molecular and biochemical differences to be more reliably attributed to disease status rather than demographic variability.

### 3.2. Laboratory, Biochemical, and Clinical Characteristics

HCC patients exhibited significant alterations in several laboratory parameters in comparison to healthy controls (Table 1). Hematological analysis showed a notable reduction in platelet count (*p* < 0.001) alongside elevated white blood cell count and hemoglobin (*p* < 0.001), a finding consistent with portal hypertension and hypersplenism associated with chronic liver disease. Serum creatinine levels were modestly higher in HCC patients (*p* < 0.001), whereas fasting glucose levels did not differ significantly between groups (*p* = 0.348).

Markers of hepatocellular injury and cholestasis, including ALT, AST, and ALP, were markedly increased in HCC patients compared to controls (*p* < 0.001 for all), reflecting hepatic injury and biliary dysfunction. Additionally, serum albumin, total bilirubin, and PC were significantly altered in HCC patients (*p* < 0.001), signifying impaired hepatic synthetic and excretory function. AFP levels were considerably elevated in the HCC group relative to controls (*p* < 0.001), supporting its established role as a conventional biomarker for HCC. All HCV RNA quantifications were negative in the control group, confirming the absence of infection, while the median viral load in the HCC cohort was 248,834.5 copies, establishing viral activity in the studied cohort. These findings collectively affirm the presence of profound biochemical disturbances in HCC patients and provide an appropriate pathological context for evaluating the diagnostic and clinical relevance of the studied lncRNAs.

The assessment of liver fibrosis revealed a predominance of early to intermediate stages, reflecting a spectrum of chronic liver injury without progression to severe cirrhosis. Regarding hepatic steatosis, 58% of patients showed no steatosis, while mild and moderate steatosis were present in 39.0% and 3.0% of cases, respectively. According to the BCLC staging system, patients were distributed across all disease stages, allowing for a meaningful evaluation of lncRNA expression across different stages of tumor progression.

### 3.3. Expression Profile of lncRNAs in HCC

**Figure 1:**
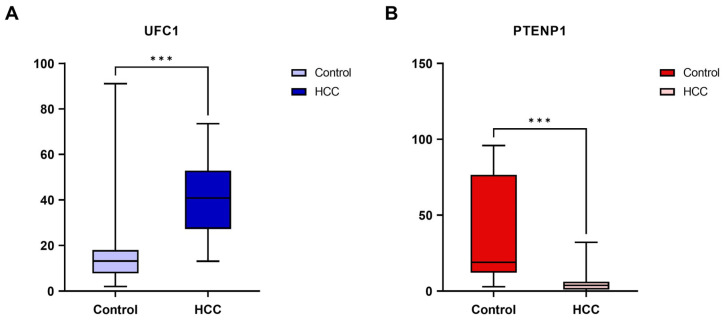
Relative expression levels of (**A**) UFC1 and (**B**) PTENP1 in hepatocellular carcinoma HCC patients and controls. *** Significant difference with *p* < 0.001.

A pronounced differential expression of the studied lncRNAs was detected between groups. UFC1 was significantly overexpressed in HCC patients compared to controls (median [IQR]: 40.86 [27.23–52.92] vs. 13.19 [7.75–18.04]; *p* < 0.001). In contrast, PTENP1 showed marked downregulation in HCC (median [IQR]: 3.78 [1.06–6.12] vs. 19.02 [12.11–76.60]; *p* < 0.001) (Figure 1). Individual data points are provided in Supplementary Figure S1.

### 3.4. Diagnostic Performance of the Studied lncRNAs

**Figure 2:**
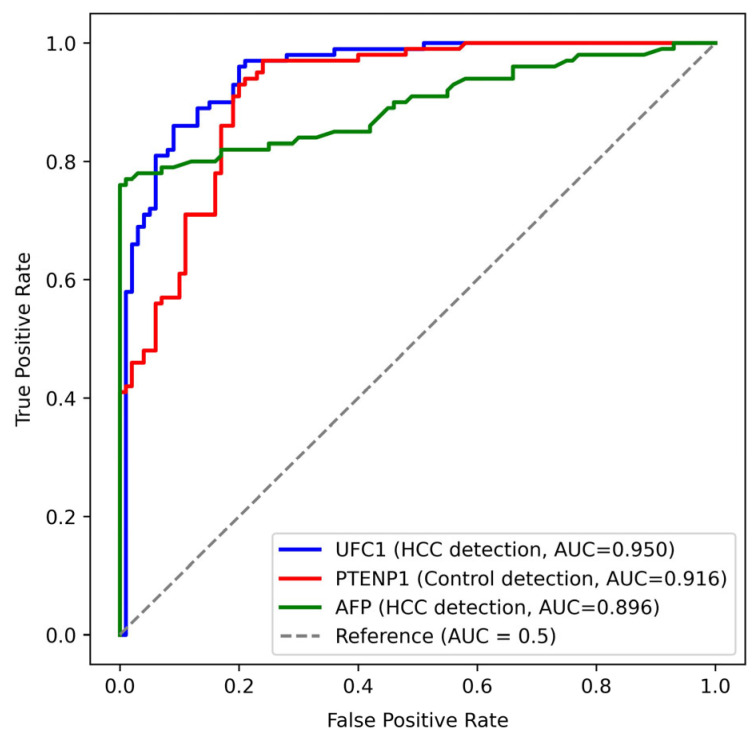
Receiver operating characteristic (ROC) curves of UFC1, PTENP1, and AFP for discriminating hepatocellular carcinoma (HCC) from controls. AUC: Area under the curve; AFP: Alpha-fetoprotein.

ROC curve analysis demonstrated substantial diagnostic accuracy for both UFC1 and PTENP1 in discriminating HCC patients from healthy controls. UFC1 achieved an AUC of 0.950 (95% CI: 0.921–0.979, *p* < 0.001), with a sensitivity of 96% and a specificity of 80% at a cutoff value of ≥19.54. PTENP1 attained an AUC of 0.916 (95% CI: 0.878–0.953, *p* < 0.001), with a sensitivity and a specificity of 93% and 80%, respectively, at a cutoff value of ≤8.37. On the other side, AFP exhibited inferior diagnostic performance, with an AUC of 0.896 (95% CI: 0.849–0.942, *p* < 0.001), a sensitivity of 82%, and a specificity of 83% at a cutoff value of ≥12.63 (Figure 2).

To evaluate whether combining markers improves diagnostic accuracy, logistic regression was used to generate predicted probabilities for two models: (1) UFC1 + PTENP1, and (2) UFC1 + PTENP1 + AFP. The UFC1 + PTENP1 model yielded an AUC of 0.977 (95% CI: 0.956–0.992, *p* < 0.001), with a sensitivity of 93% and specificity of 96% at a cutoff of 0.536. The UFC1 + PTENP1 + AFP model produced an AUC of 0.991 (95% CI: 0.980–0.998, *p* < 0.001), with a sensitivity of 98% and specificity of 96% at a cutoff of 0.407. The bootstrap analysis showed that the addition of AFP resulted in a statistically significant improvement in AUC (mean ΔAUC = 0.0140, 95% CI: 0.0045–0.0276, *p* < 0.001), indicating that the three-marker panel offers the highest diagnostic accuracy.

### 3.5. Correlation of lncRNAs with Clinical and Laboratory Parameters

**Figure 3:**
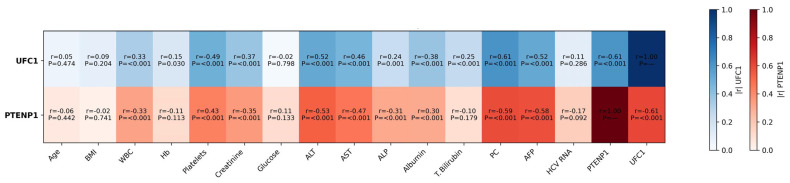
Correlation matrix illustrating associations between UFC1, PTENP1, and clinical and laboratory parameters in the Study Cohort. BMI: Body mass index; WBCs: White blood cells; Hb: Hemoglobin; ALT: Alanine aminotransferase; AST: Aspartate aminotransferase; ALP: Alkaline phosphatase; T. bilirubin: Total bilirubin; PC: Prothrombin concentration; AFP: Alpha-fetoprotein.

Spearman correlation analysis revealed significant associations between lncRNA expression levels and multiple clinical and laboratory variables. The expression of UFC1 showed positive correlations with WBC count, ALT, AST, ALP, PC, creatinine, and AFP (*p* < 0.001 for all), and a negative correlation with platelet count and albumin (*p* < 0.001). PTENP1 expression exhibited inverse relationships with almost the same set of parameters (*p* < 0.001 for most). A strong, statistically significant inverse correlation was observed between UFC1 and PTENP1 (r = −0.609, *p* < 0.001), suggesting an antagonistic regulatory relationship between these lncRNAs in HCC pathogenesis. No significant correlations were found with age, BMI, or glucose levels (Figure 3).

### 3.6. Association of lncRNA Expression with Fibrosis, Steatosis, and BCLC Stage

**Figure 4:**
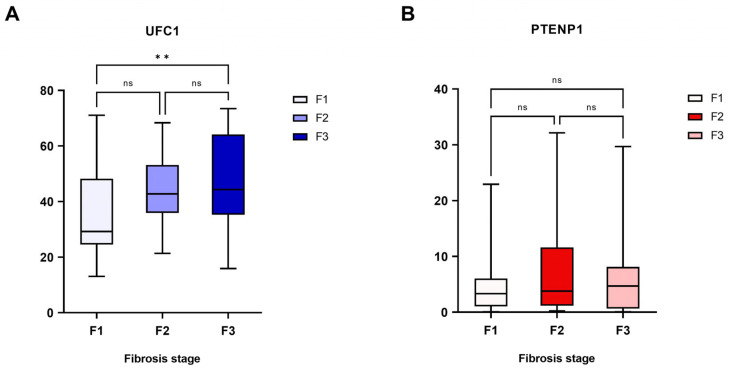
Expression levels of (**A**) UFC1 and (**B**) PTENP1 across different grades of fibrosis in hepatocellular carcinoma (HCC) patients. ** Significant difference with *p* < 0.01. ns: non-significant difference.

Analysis of lncRNA expression across fibrosis stages indicated a positive relationship between UFC1 expression and fibrosis progression (*p* = 0.005), with a trend toward higher levels in advanced stages. However, given the observed within-group variability, this finding should be interpreted with caution and warrants further investigation in larger cohorts. Conversely, PTENP1 expression did not significantly correlate with fibrosis grade (Figure 4; Individual data points are provided in Supplementary Figure S2). Neither UFC1 nor PTENP1 expression was significantly associated with hepatic steatosis grades, denoting that their dysregulation is more closely linked to fibrotic and oncogenic processes than lipid accumulation.

**Figure 5:**
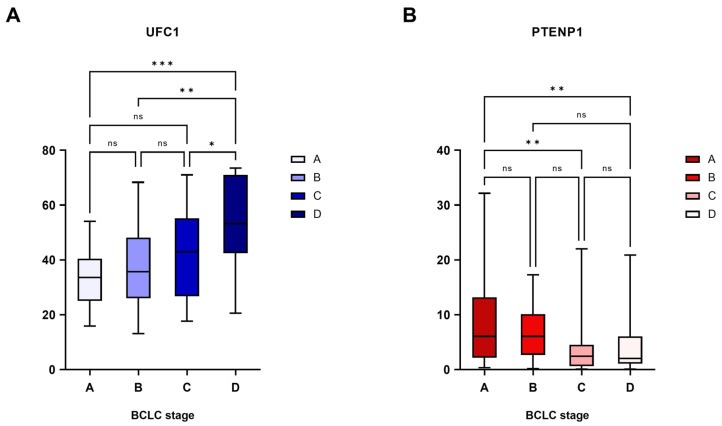
Expression levels of (**A**) UFC1 and (**B**) PTENP1 across different BCLC Stages in hepatocellular carcinoma (HCC) patients. Panels on the x-axis represent BCLC stages A, B, C, and D. *** Significant difference with *p* < 0.001. ** Significant difference with *p* < 0.01. * Significant difference with *p* < 0.05. ns: non-significant difference.

Both lncRNAs displayed substantial correlations with BCLC stage. UFC1 expression increased progressively with advancing tumor stage (*p* < 0.001), whereas a decline in PTENP1 expression was observed from early to advanced stages (*p* = 0.003) (Figure 5; Individual data points are provided in Supplementary Figure S3). Despite the statistical significance, the distribution of individual values across stages exhibited considerable overlap, underscoring the need for cautious interpretation and further validation.

### 3.7. Prognostic Performance of the Studied lncRNAs

**Figure 6:**
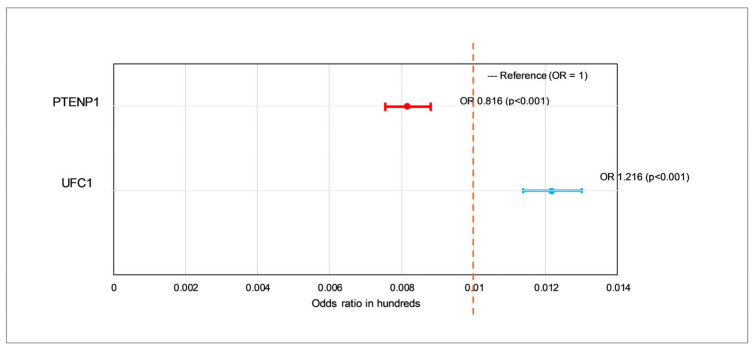
Logistic regression analysis of UFC1 and PTENP1 as predictors of hepatocellular carcinoma (HCC) risk.

Logistic regression analysis implied that both UFC1 and PTENP1 were independent and statistically significant predictors of HCC status. Elevated UFC1 expression was positively associated with HCC, with each unit increase corresponding to a 21.6% higher odds of disease occurrence (OR = 1.216, 95% CI: 1.137–1.300; *p* < 0.001), supporting its proposed oncogenic role. On the contrary, PTENP1 expression demonstrated a notable inverse relationship with HCC, where each unit increase resulted in a reduced likelihood of illness (OR = 0.816, 95% CI: 0.756–0.880; *p* < 0.001), consistent with its tumor-suppressive function (Figure 6).

## 4. Discussion

The present study investigated the expression patterns and clinical significance of two biologically antagonistic lncRNAs, UFC1 and PTENP1, in HCC. It was found that UFC1 was significantly upregulated (~2.9-fold), whereas PTENP1 was markedly downregulated (~4-fold) in HCC patients compared with healthy controls. This opposing pattern suggests distinct roles in hepatocarcinogenesis, positioning UFC1 as a potential oncogene and PTENP1 as a tumor suppressor. They are not only strongly associated with the disease state but also correlate with key clinical and pathological hallmarks of HCC progression. Importantly, the differences in their expression were independent of demographic confounders, due to successful matching of our cohorts, supporting a disease-specific alteration in lncRNA expression.

Our findings for UFC1 align with previous studies, which revealed that its upregulation promotes HCC progression, reduces apoptosis, and correlates with tumor size, BCLC stage, and unfavorable patient outcomes [13,15]. It acts as a competing endogenous RNA (ceRNA) that sequesters tumor-suppressive miRNAs like miR-34a, leading to the activation of oncogenic pathways, including Wnt/β-catenin. It also controls the expression of β-catenin through the binding to HuR, an RNA-binding protein that subsequently interacts with β-catenin mRNA [13,15]. Similarly, a prior study reported that UFC1 was overexpressed in colorectal cancer (CRC) patient tissues and exhibited a positive correlation with tumor stage, while its knockdown suppressed proliferation by downregulating cyclin D1 and induced apoptosis of CRC cells through the inhibition of β-catenin and stimulation of p38 signaling [16]. In breast cancer, UFC1 was found to promote tumor proliferation and migration through the miR-34a/CXCL10 axis [17]. Also, UFC1 was elevated in gastric cancer (GC) and contributed to its progression and invasion by functioning as a miR-498 sponge to activate Lin28b expression [12]. In addition, Zhang et al. demonstrated that UFC1 expression level was increased in non-small cell lung cancer (NSCLC) tumor tissues, serum, and exosomes of patients, with high levels linked to tumor infiltration [18]. They showed that exosome-transmitted UFC1 could bind to EZH2 to repress PTEN expression and activate PI3K/Akt signaling, hence enabling NSCLC tumorigenesis. Moreover, blood levels of UFC1 expression were considerably greater in patients with pancreatic cancer than in healthy controls and corresponded to lymph nodes involvement, metastases to distant organs, and clinical stage [19]. These mechanistic insights, derived from previous functional studies, provide a plausible framework; however, our data alone do not establish causality in the context of HCC.

PTENP1 is a well-characterized tumor-suppressive pseudogene lncRNA, known to negatively regulate the oncogenic PTEN/AKT/mTOR pathway by serving as a decoy for miRNAs that target the PTEN tumor suppressor gene [20]. In this study, the tumor-suppressive role of PTENP1 is evidenced by its strong inverse correlations with the clinical severity parameters and its progressive downregulation across advancing BCLC stages, which further suggests that loss of PTENP1 expression may facilitate disease progression. In accordance with our results, the overexpression of PTENP1 prompts cell cycle arrest, senescence, autophagy, and apoptotic cell death in cancer cells [21].

A pivotal contribution of this study is the superior diagnostic performance of both lncRNAs compared to the conventional biomarker AFP. The AUC values for UFC1 (0.950) and PTENP1 (0.916) surpassed that of AFP (0.896), with both lncRNAs offering higher sensitivity (96% and 93%, respectively). This implies that UFC1 and PTENP1 hold significant promise as sensitive blood-based biomarkers for the identification of HCC. The strong inverse correlation (r = −0.609) between their expression levels hints at a possible antagonistic regulatory network, where the rise of an oncogenic lncRNA (UFC1) coincides with the suppression of a tumor-suppressive one (PTENP1), a dynamic that could be central to HCC development. The diagnostic performance of both markers exceeds that frequently documented for other well-studied oncogenic lncRNAs in HCC, including HULC, Linc00152, and MALAT1, whose AUC values range between 0.57 and 0.85 [22,23,24]. In addition, combining UFC1 and PTENP1 into a single diagnostic model yielded a strong AUC of 0.977. Adding AFP to this panel further improved diagnostic accuracy to an AUC of 0.991, with a bootstrap-confirmed significant increase in sensitivity (from 93% to 98%). These results underscore the potential of integrating UFC1 and PTENP1 into existing surveillance paradigms, either as a stand-alone liquid biopsy or in combination with AFP for enhanced detection.

The clinical relevance of these biomarkers is underscored by their substantial correlations with disease severity. The expression of UFC1 showed significant positive relationships with markers of liver injury (ALT and AST), tumor burden (AFP), and synthetic dysfunction. Notably, its level escalated with progressing BCLC stages and fibrosis grades, peaking in BCLC D-stage and F3-stage, thereby positioning it as a potential marker of tumor progression and hepatic damage. This novel insight indicates that UFC1 may be involved in the activation of fibrogenic signaling pathways and early fibrotic microenvironment remodeling. Although PTENP1 expression did not correlate with fibrosis, its noticeable decline with advancing BCLC stage reinforces its link to tumor aggressiveness rather than the underlying cirrhotic process alone. The lack of association with hepatic steatosis for both lncRNAs suggests that their dysregulation is more specific to fibrogenic and oncogenic pathways rather than metabolic liver injury.

Logistic regression analysis confirmed that UFC1 and PTENP1 are independent predictors of HCC status, further supporting their potential clinical utility. Elevated UFC1 expression was associated with a 21.6% higher odds of disease per unit increase (OR = 1.216). In contrast, each unit increase in PTENP1 expression resulted in diminished likelihood of illness (OR = 0.816). These results position UFC1 as a candidate risk factor and PTENP1 as a potential protective factor, highlighting their roles in the disease mechanism.

While the overall diagnostic performance of UFC1 and PTENP1 was very promising, a small number of healthy controls showed expression levels overlapping with those of HCC patients. Such outliers may reflect subclinical liver pathology, which could influence lncRNA expression even in the absence of overt malignancy. Alternatively, they may represent biological variability inherent to circulating RNA measurements. To minimize false-positive results in clinical application, we recommend using the combined panel of UFC1, PTENP1, and AFP, which achieved a higher specificity (96%) and interpreting these markers in conjunction with clinical and radiological evaluation. Prospective studies with longitudinal follow-up will be valuable to determine whether outliers in the control group subsequently develop liver disease.

An important consideration for the clinical translation of circulating lncRNAs like UFC1 and PTENP1 is their stability in bodily fluids, which is influenced by several factors. Circulating lncRNAs are protected from RNase degradation through encapsulation within extracellular vesicles such as exosomes, or by forming complexes with high-density lipoproteins or RNA-binding proteins, e.g., protein Argonaute 2 [25,26]. This structural packaging not only ensures their stability but also facilitates their intercellular communication and functional roles in recipient cells. The differential partitioning of specific lncRNAs into these various carriers (e.g., exosome-bound vs. protein-bound) can influence their detectability and measured abundance by qPCR. For instance, exosome-transmitted UFC1 has been shown to mediate intercellular signaling and inhibit PTEN expression in non-small cell lung cancer, promoting its progression [18]. This suggests that the fraction of an lncRNA within exosomes might hold specific pathological significance. Our study measured total RNA from whole blood, capturing both vesicle-encapsulated and protein-bound lncRNA fractions. While our findings demonstrate strong diagnostic and prognostic associations, future studies should explore the relative contributions of these distinct circulating subpopulations to fully understand the biology and optimize the detection methods for UFC1 and PTENP1 as robust liquid biopsy biomarkers.

A valuable aspect for understanding the functional roles of UFC1 and PTENP1 is their cellular localization. In the circulation, these lncRNAs likely originate from a combination of tumor cells, surrounding non-malignant hepatocytes, infiltrating immune cells, and exosomal vesicles [27]. Future studies employing RNA fluorescence in situ hybridization (FISH) or single-cell RNA sequencing would be valuable to precisely map the cellular sources of these lncRNAs in normal liver and HCC tissues, further elucidating their contributions to hepatocarcinogenesis.

Several limitations of this study warrant consideration. First, the single-center and moderate sample size necessitate validation in larger, multi-ethnic cohorts. Second, our control group consisted of healthy individuals rather than patients with cirrhosis or chronic liver disease. Future studies should evaluate the performance of UFC1 and PTENP1 in high-risk populations to better reflect real-world clinical scenarios. Third, the cross-sectional nature allows for the establishment of association but not causality. While the correlations with disease stage are compelling, longitudinal studies are necessary to ascertain whether UFC1 and PTENP1 can predict clinical outcomes, including survival or treatment response. Finally, although our results strongly demonstrate biological relevance, further in vitro and in vivo functional studies are required to fully elucidate the precise molecular mechanisms governing the roles of these lncRNAs in HCC pathogenesis.

## 5. Conclusions

This study identifies UFC1 and PTENP1 as powerfully dysregulated lncRNAs in HCC in an Egyptian cohort. Their promising diagnostic accuracy and significant correlations with disease severity, particularly BCLC stage, underscore their potential dual utility as novel non-invasive biomarkers for detection and as indicators of pathological progression. While the findings are compelling, the observed variability within groups warrants cautious interpretation and highlights the need for further validation in high-risk populations (e.g., patients with cirrhosis or chronic liver disease) and larger, multi-center cohorts. These results merit further investigation for their integration into refined diagnostic panels and exploration as therapeutic targets in HCC management.

## Figures and Tables

**Table 1:** Baseline demographic, laboratory, and clinical characteristics of hepatocellular carcinoma (HCC) patients and controls.

		Control (*n* = 100)	HCC (*n* = 100)	*p*-Value
**Age (years)**		41.42 ± 0.84	42.71 ± 0.95	0.308
**Gender**				
Females		30 (30.0%)	35 (35.0%)	0.450
Males		70 (70.0%)	65 (65.00%)	
**BMI**		25.84 ± 0.34	26.67 ± 0.36	0.089
**Laboratory data**				
WBCs (×1000/µL)		4.87 ± 0.15	6.54 ± 0.18	<0.001
Hemoglobin (g/dL)		12.81 ± 0.16	13.64 ± 0.16	<0.001
Platelets (×1000/µL)		322.45 ± 4.60	225.05 ± 5.96	<0.001
ALT (IU/L)		19.10 (12.98–23.55)	42.50 (26.50–67.75)	<0.001
AST (IU/L)		21.90 (17.13–27.48)	39.00 (28.0–56.75)	<0.001
ALP (IU/L)		66.50 (56.35–77.6)	91.00 (70.00–120.75)	<0.001
Albumin (g/dL)		4.24 ± 0.05	3.79 ± 0.04	<0.001
Total Bilirubin (mg/dL)		0.60 ± 0.11	0.75 ± 0.03	<0.001
PC (%)		69.57 ± 0.71	92.23 ± 0.98	<0.001
Creatinine (mg/dL)		0.70 ± 0.02	0.90 ± 0.02	<0.001
Glucose (mg/dL)		100.52 ± 2.08	97.74 ± 2.10	0.348
AFP (ng/mL)		7.3 (4.15–10.36)	64.90 (19.63–174.22)	<0.001
Quantitative HCV RNA (copies)		0	248,834.5 (46,025–591,431.75)	N.A
**Clinical data**				
Fibrosis Grade	F1		45 (45.0%)	
	F2		20 (20.0%)	
	F3		35 (35.0%)	
Steatosis	None		58 (58.0%)	
	Mild		39 (39.0%)	
	Moderate		3 (3.0%)	
BCLC Stage	A		24 (24.0%)	
	B		23 (23.0%)	
	C		30 (30.0%)	
	D		23 (23.0%)	

Parametric variables are represented as mean ± SE. Non-parametric variables are represented as median and interquartile range (25–75%). HCC: Hepatocellular carcinoma; BMI: Body mass index; WBCs: White blood cells; ALT: Alanine aminotransferase; AST: Aspartate aminotransferase; ALP: Alkaline phosphatase; PC: Prothrombin concentration; AFP: Alpha-fetoprotein.

## Data Availability

The original contributions presented in this study are included in the article/Supplementary Material. Further inquiries can be directed to the corresponding author.

## Supplementary Materials

The following supporting information can be downloaded at https://www.mdpi.com/article/10.3390/cimb48040360/s1.

## Abbreviations

The following abbreviations are used in this manuscript:

AFP	Alpha-fetoprotein
ALP	Alkaline phosphatase
ALT	Alanine aminotransferase
AST	Aspartate aminotransferase
AUC	Area under the curve
BCLC	Barcelona Clinic Liver Cancer staging system
BMI	Body mass index
CRC	Colorectal cancer
HBV	Hepatitis B virus
HCC	Hepatocellular carcinoma
HCV	Hepatitis C virus
lncRNAs	Long non-coding RNAs
NSCLC	Non-small cell lung cancer
OR	Odds ratio
PC	Prothrombin concentration
PTENP1	Phosphatase and tensin homolog pseudogene 1
UFC1	Ubiquitin-fold modifier conjugating enzyme 1
