# Supplementary material for: Novel lncRNA Signature (UFC1/PTENP1) as a Molecular Biomarker for the Diagnosis and Prognosis of Hepatocellular Carcinoma in an Egyptian Cohort
Source: Curr Issues Mol Biol. 2026 Mar 29;48(4):360. doi: 10.3390/cimb48040360 (PMC13114740; doi:10.3390/cimb48040360)
Supplement: Supplementary file 1 [file cimb-48-00360-s001.zip › cimb-4222467-supplementary.pdf]

Novel lncRNA Signature (UFC1/PTENP1) as a Molecular Biomarker for the Diagnosis and Prognosis of Hepatocellular Carcinoma in an Egyptian Cohort

Marwa Hassan [1,*], Lobna Abdelsalam [2], Amal Kotb Behery [2] and Rania Fathy Elnahas [2]

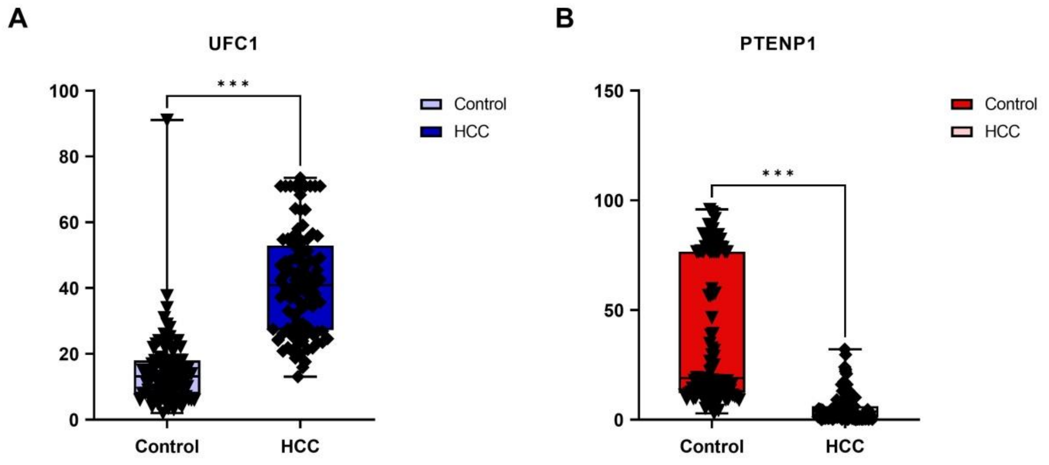

Figure S1. Relative expression levels of (A) UFC1 and (B) PTENP1 in hepatocellular carcinoma HCC patients and controls. ***Significant difference with $p<0.001$.

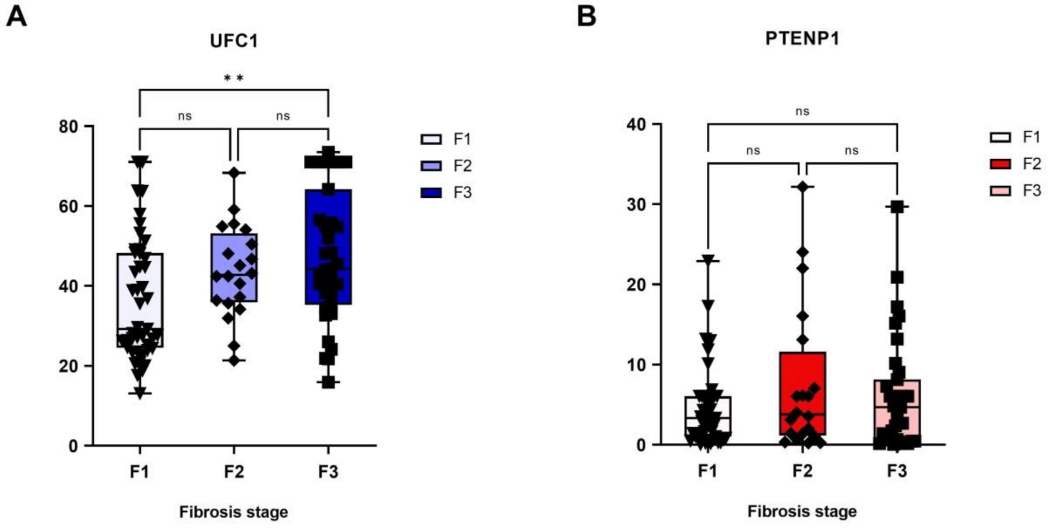

Figure S2: Expression levels of (A) UFC1 and (B) PTENP1 across different grades of fibrosis in hepatocellular carcinoma (HCC) patients. **Significant difference with $p<0.01$. ns: non-significant difference.

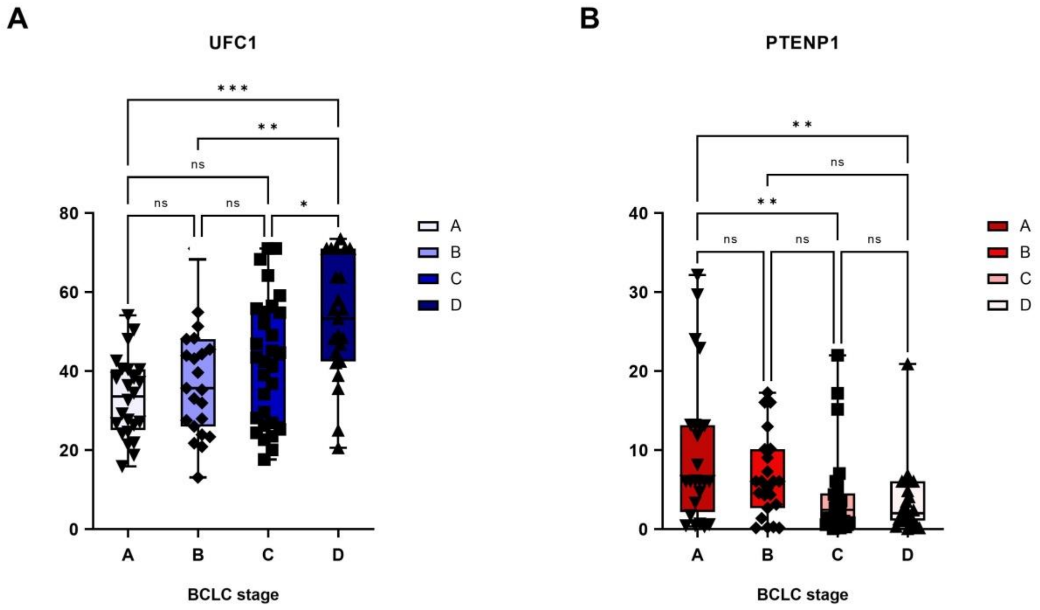

Figure S3: Expression levels of (A) UFC1 and (B) PTENP1 across different BCLC Stages in hepatocellular carcinoma (HCC) patients. Panels on the x-axis represent BCLC stages A, B, C, and D. ***Significant difference with *p*<0.001. **Significant difference with *p*<0.01. *Significant difference with *p*<0.05. ns: non-significant difference.
